# Effects of exenatide twice daily versus sitagliptin on 24-h glucose, glucoregulatory and hormonal measures: a randomized, double-blind, crossover study

**DOI:** 10.1111/j.1463-1326.2011.01428.x

**Published:** 2011-11

**Authors:** J K Berg, S K Shenouda, C R Heilmann, A L Gray, J H Holcombe

**Affiliations:** 1 Cetero ResearchSan Antonio, TX, USA; 2 Lilly USA, LLCIndianapolis, IN, USA

**Keywords:** *β* cell, clinical trial, diabetes mellitus, DPP-IV inhibitor, GLP-1 analogue, incretins

## Abstract

**Aim:** To compare exenatide and sitagliptin glucose and glucoregulatory measures in subjects with type 2 diabetes.

**Methods:** An 8-week, double-blind, randomized, crossover, single-centre study. Eighty-six subjects (58% female, body mass index 35 ± 5 kg/m^2^, haemoglobin A1c 8.3 ± 1.0%) received either exenatide 10 µg (subcutaneous) twice daily or sitagliptin 100 mg (oral) daily for 4 weeks and crossed to the other therapy for an additional 4 weeks. Main outcome was time-averaged glucose during the 24-h inpatient visits.

**Results:** Both treatments decreased average 24-h glucose, but exenatide had a greater effect [between-group difference: −0.67 mmol/l, 95% confidence interval (CI): −0.9 to −0.4 mmol/l]. Both treatments decreased 2-h postprandial glucose (PPG), area under the curve of glucose above 7.8 mmol/l (140 mg/dl) and 11 mmol/l (200 mg/dl) and increased the time spent with glucose between 3.9 and 7.8 mmol/l (70 and 140 mg/dl) during 24 h, but exenatide had a significantly greater effect (p < 0.05). Both treatments decreased postprandial serum glucagon, with exenatide having a greater effect (p < 0.005). Both treatments decreased fasting blood glucose to a similar degree (p = 0.766). Sitagliptin increased, while exenatide decreased, postprandial intact glucagon-like peptide-1. Both drugs improved homeostasis model assessment of *β*-cell function (HOMA-B), with exenatide having a significantly greater effect (p = 0.005). Both exenatide and sitagliptin decreased 24-h caloric intake, with exenatide having a greater effect (p < 0.001). There was no episode of major hypoglycaemia. Adverse events were mild to moderate and mostly gastrointestinal in nature with exenatide. No study withdrawals were due to an adverse event.

**Conclusion:** Compared to sitagliptin, exenatide showed significantly lower average 24-h glucose, 2-h PPG, glucagon, caloric intake and improved HOMA-B.

## Introduction

Advances in the treatment of hyperglycaemia include glucagon-like peptide-1 (GLP-1) receptor agonists, such as exenatide and liraglutide, and dipeptidyl peptidase-4 (DPP-4) inhibitors, such as sitagliptin and saxagliptin. Exenatide binds and activates the GLP-1 receptor, while sitagliptin increases endogenous GLP-1 concentrations by inhibiting DPP-4 degradation of circulating GLP-1 [1,2]. Exenatide stimulates glucose-dependent insulin secretion, suppresses inappropriately high glucagon secretion, slows gastric emptying and reduces food intake [3–7]. Sitagliptin enhances glucose-dependent insulin secretion and decreases glucagon secretion with no significant effect on gastric emptying or food intake [8,9].

A recent clinical study by DeFronzo et al. [10] comparing the acute effects of exenatide twice daily (BID) and sitagliptin reported that exenatide produced a greater decrease in 2-h postprandial glucose (PPG) than sitagliptin. The primary objective of the current study was to examine glucose profiles over an entire 24-h period in subjects treated with exenatide or sitagliptin and to compare their mechanisms of action using different measures of glycaemic control, *β*-cell function, *α*-cell function and 24-h caloric intake.

## Methods

### Experimental Design

This was an 8-week, 2-arm, 2-period crossover, double-blind, double-dummy, randomized, active comparator trial. The study was approved by the institutional review board at one site in the USA that enrolled patients with type 2 diabetes into the study from 2008 to 2009. Inclusion criteria were age 18–70 years, body mass index (BMI) 25–45 kg/m^2^, stable body weight for at least 3 months prior to screening visit, haemoglobin A1c (HbA1c) ≥7% and ≤11%, fasting glucose <280 mg/dl and subjects must have been on a stable dose of metformin or thiazolidinedione for at least 60 or 120 days, respectively. Exclusion criteria were females of childbearing potential; treatment with insulin, exenatide (or any GLP-1 receptor agonist), sulphonylureas, drugs affecting gastrointestinal motility, weight loss drugs, corticosteroids, *α*-glucosidase inhibitors; history of organ transplant; history of liver or renal disease; fasting triglycerides >400 mg/dl; blood pressure >165/90 mm Hg. All subjects provided written informed consent prior to undergoing any study procedure or receiving any study treatment. The study was performed in accordance with the principles of the Declaration of Helsinki [11] and all regulatory requirements.

At the baseline and subsequent two inpatient stays, subjects were admitted during late afternoon (17:00 hours) for 24 h. Following the baseline visit, all subjects began self-administering study treatments at home. All subjects received a capsule once daily in the morning (sitagliptin 100 mg or placebo) and two injections [exenatide 5 µg (1st week) to 10 µg (additional 3 weeks), or placebo] in addition to their prestudy medication(s). At the end of the first 4 weeks, subjects were switched to the alternative treatment. Subjects received their injections and a capsule before the breakfast test meal during the postrandomization 24-h inpatient visits.

The primary efficacy measure was the time-averaged glucose during the 24-h inpatient visits [12]. The 24-h mean glucose was calculated from 36 glucose measurements over 24 h. Additional efficacy measures were fasting glucose (08:00 hours); 2-h PPG (from start of breakfast meal); difference between minimum and maximum glucose concentrations during 24 h calculated from highest glucose value to lowest glucose value; area under the curve (AUC) of glucose above 7.8 mmol/l [AUC > 7.8 mmol/l (140 mg/dl)] and above 11 mmol/l [AUC > 11 mmol/l (200 mg/dl)] calculated by the trapezoid method, using area above 7.8 or 11 mmol/l, respectively. The WHO criteria for the diagnosis of diabetes and impaired glucose tolerance, as well as previous work [12], were considerations in the choice of these arbitrary values to put the hyperglycaemic exposure into a clinical context; proportion of time during 24 h with a glucose 3.9–7.8 mmol/l; and homeostasis model assessment of *β*-cell function (HOMA-B), calculated using the updated HOMA model [13]. Safety data collected included hypoglycaemic and other adverse events. Minor hypoglycaemia was defined as self-reported transient symptoms of hypoglycaemia and a blood glucose <3 mmol (54 mg/dl). Major hypoglycaemia was defined as any episode consistent with hypoglycaemia resulting in the loss of consciousness or seizure or documented hypoglycaemia <3 mmol (54 mg/dl) requiring assistance. All efficacy and safety measures were predefined in the study protocol. Because of the short duration of this study, HbA1c was measured only at baseline.

### Individualized Meal and Caloric Intake Measurements

During the 24-h inpatient visits, three similar caloric and macronutrient content meals were given, beginning with the evening meal. All meals were individualized for each subject based on gender and weight [10,12]. The mixed test meal (breakfast) provided approximately 7 kcal/kg (55% carbohydrate, 15% protein and 30% fat). Subjects were encouraged to consume the entire breakfast meal within 15 min. Any calories not consumed were carried over to the midday meal. Subjects had no time restriction for the midday or evening meals. Food intake was recorded by weighing the food not consumed at each meal.

### Statistical Analyses

Statistical analyses were performed using SAS version 9.1. All analyses were based on two-sided tests at the 0.05 *α* level. To account for multiplicity, p-values for postbreakfast serial measurements were reported only if <0.005 (0.05/10) instead of <0.05, as there were 10 or fewer values for each measurement. Baseline characteristics were described by mean, standard deviation (SD) or n (%) and postbaseline efficacy values were described by least-squares (LS) mean and standard error (SE).

Analysis of efficacy variables and hypoglycaemia included all subjects who received at least one dose of study medication and completed the first treatment period. Baseline for both treatment periods was defined as the randomization visit. Adverse events were analyzed on all randomized subjects. Analyses for continuous variables used Grizzle's model [14], including effects for treatment, period, sequence, baseline of the variable analyzed and baseline HbA1c stratum (<8.5%, ≥8.5%), with subject as a random effect. Assuming a treatment difference of 0.5 mmol/l and a within-patient SD of 1.1 mmol/l, 58 completing subjects would provide 90% power to detect a significant difference between the average 24-h glucose concentrations during treatment with exenatide or sitagliptin.

## Results

### Subject Characteristics

**Figure 1:**
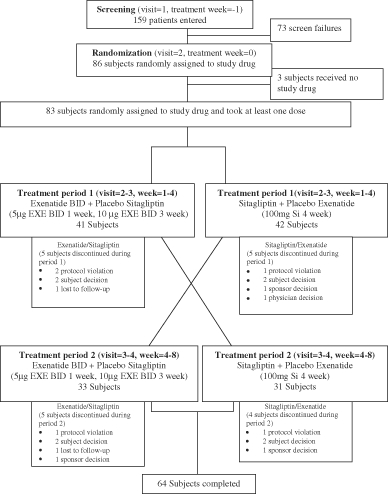
Study design and subject disposition. Subjects were to inject 5 µg (BID, before morning and evening meals) of exenatide during the first week of the treatment period. Thereafter, subjects were to inject 10 µg (BID, before morning and evening meals) of exenatide for the remainder of the treatment period. Placebo exenatide was administered in the same manner. Subjects were further randomized to administer their exenatide or placebo injection either within 15 min before meals or 45–60 min before meals. Subjects continued to administer injections at their specified time throughout the study except during the 24-h assessments. BID, twice daily; EXE, exenatide; QAM, once daily in the AM; Si, sitagliptin.

Eighty-six subjects were randomized but three subjects discontinued before receiving the study drug. Of those 83 subjects, 82 were receiving metformin and 1 was receiving thiazolidinedione (figure 1). The treatment sequences were exenatide/sitagliptin n = 41 and sitagliptin/exenatide n = 42 (figure 1). Baseline characteristics are shown in Table 1.

**Table 1:** Subject demographics and baseline characteristics for exenatide/sitagliptin and sitagliptin/exenatide sequences

	Exenatide/sitagliptin sequence (n = 41)	Sitagliptin/exenatide sequence (n = 42)	Overall
Age (years)	55 ± 10	54 ± 9	54 ± 10
Sex: females, n (%)	19 (46)	29 (69)	48 (58)
Race, n (%)			
American Indian/Native American	1 (2)	0 (0)	1 (1)
Black/African American	2 (5)	3 (7)	5 (6)
Caucasian	38 (93)	39 (93)	77 (93)
Ethnicity, n (%)			
Hispanic or Latino	25 (61)	30 (71)	55 (66)
Non-Hispanic or Latino	16 (39)	12 (29)	28 (34)
Body weight (kg)	98.2 ± 22.5	94.0 ± 20.3	96.1 ± 21.4
Height (cm)	166.6 ± 10.6	163.7 ± 10.3	165.2 ± 10.5
BMI (kg/m^2^)	35.0 ± 5.5	34.9 ± 5.5	34.9 ± 5.5
Duration of diabetes (years)	7 ± 6	8 ± 7	7 ± 7
HbA1c (%)	8.3 ± 1.0	8.3 ± 1.1	8.3 ± 1.0
Fasting TG (mmol/l)	2.1 ± 1.0	3.4 ± 8.2	2.8 ± 5.9

BMI, body mass index; HbA1c, haemoglobin A1c; n, number of subjects; TG, triglycerides.

### 24-Hour Glucose Profiles

**Figure 2:**
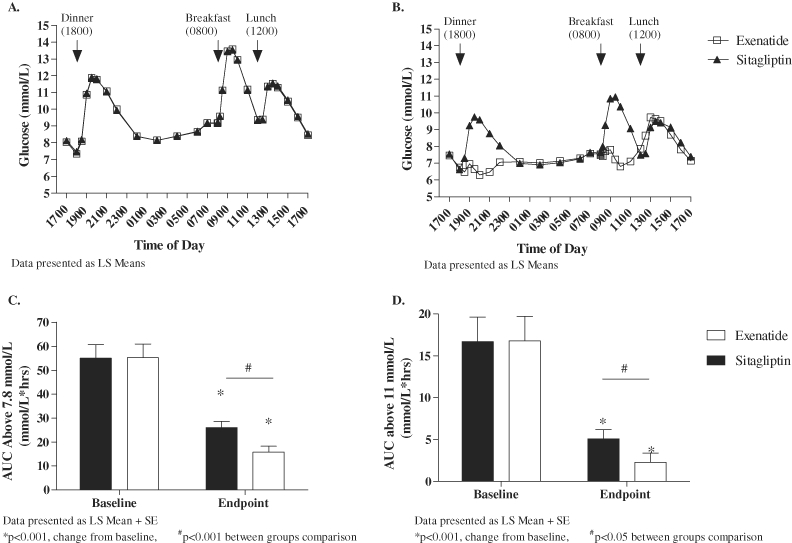
Metabolic parameters. (A) 24-h glucose profile at baseline; (B) 24-h glucose profile at endpoint; (C) AUC for glucose values above 7.8 mmol/l and (D) AUC for glucose values above 11 mmol/l. AUC, area under the curve.

At baseline, the 24-h glucose profiles were similar between treatment groups (figure 2A). After 4 weeks, both treatments showed a significant (p < 0.001) reduction from baseline in the average 24-h glucose. Exenatide treatment led to a greater reduction (p < 0.001) than sitagliptin from baseline in average glucose concentration [between-group difference: −0.67 mmol/l, 95% CI: −0.9, −0.4 mmol/l] over 24 h (figure 2B, Table 2).

**Table 2:** Baseline, endpoint and change in the metabolic parameters, *β*-cell function and blood pressure

	Exenatide			Sitagliptin			
			
	LS mean (s.e.m.)	p-value*	95% CI	LS mean (s.e.m.)	p-value*	95% CI	p-value†
24-h Averaged glucose (mmol/l)		<0.001			<0.001		<0.001
Baseline	9.7(0.3)			9.7(0.3)			
Endpoint	7.4(0.1)			8.1(0.1)			
Change	−2.3(0.1)		−2.6 to 2.0	−1.6(0.1)		−1.9 to −1.4	
2-h PPG (mmol/l)		<0.001			<0.001		<0.001
Baseline	12.9(0.4)			12.9(0.4)			
Endpoint	6.9(0.2)			10.5(0.2)			
Change	−6.0(0.2)		−6.5 to −5.5	−2.5(0.2)		−2.9 to −2.0	
Fasting glucose (mmol/l)		<0.001			<0.001		0.766
Baseline	9.2(0.3)			9.2(0.3)			
Endpoint	7.6(0.1)			7.6(0.1)			
Change	−1.6(0.1)		−1.9 to −1.3	−1.6(0.1)		−1.9 to −1.3	
Difference between max and min glucose (mmol/l)		<0.001			<0.001		0.010
Baseline	7.8(0.2)			7.7(0.3)			
Endpoint	5.5(0.2)			6.2(0.2)			
Change	−2.3(0.2)		−2.7 to −1.9	−1.5(0.2)		−1.9 to −1.1	
AUC for glucose >7.8 mmol/l (mmol/l × h)		<0.001			<0.001		<0.001
Baseline	55.3(5.6)			55.1(5.6)			
Endpoint	15.8(2.5)			26.1(2.5)			
Change	−39.6(2.5)		−44.6 to −34.6	−29.3(2.5)		−34.3 to −24.3	
AUC for glucose >11 mmol/l (mmol/l × h)		<0.001			<0.001		0.010
Baseline	16.8(2.9)			16.7(2.9)			
Endpoint	2.3(1.1)			5.1(1.1)			
Change	−14.5(1.1)		−16.7 to −12.4	−11.8(1.1)		−13.9 to −9.6	
Time with glucose between 3.9 and 7.8 mmol/l (h)		<0.001			<0.001		<0.001
Baseline	7.4(0.9)			7.1(0.8)			
Endpoint	15.1(0.6)			12.4(0.6)			
Change	7.9(0.6)		6.6–9.1	5.2(0.6)		4.0–6.5	
HOMA-B (%)		<0.001			<0.001		0.005
Baseline	53.2(4.2)			53.8(4.3)			
Endpoint	86.1(3.8)			74.0(3.9)			
Change	32.9(3.8)		25.4–40.4	20.8(3.9)		13.0–28.6	
Diastolic BP (mm/Hg)		0.250			0.325		0.112
Baseline	74.8(1.0)			75.4(1.1)			
Endpoint	76.2(0.9)			74.2(0.9)			
Change	1.1(0.9)		−0.8 to 2.9	−0.9(0.9)		−2.7 to 0.9	
Systolic BP (mm/Hg)		0.106			0.198		0.818
Baseline	124.6(1.8)			125.4(1.8)			
Endpoint	122.5(1.5)			123.0(1.6)			
Change	−2.5(1.5)		−5.6 to 0.5	−2.0(1.6)		−5.1 to 1.1	

AUC, area under the curve; BP, blood pressure; CI, confidence interval; HOMA-B, homeostasis model assessment of *β*-cell function; LS Mean, least-squares mean; max, maximum; min, minimum; PPG, postprandial glucose; s.e.m., standard error of the mean.

* Change from baseline.

† Between-treatment comparison.

Both exenatide and sitagliptin significantly (p < 0.001) reduced fasting glucose and mean 2-h PPG from baseline (Table 2). A significant (p < 0.001) treatment difference, favouring exenatide, was shown at endpoint for the 2-h PPG [−3.5 mmol/l, 95% CI (−4.2, −2.9 mmol/l)] but not for fasting glucose (Table 2). The difference between the minimum and maximum glucose values during the 24-h inpatient visit significantly (p < 0.001) decreased in both treatment groups with a treatment difference (p = 0.01) favouring exenatide (Table 2).

At endpoint, both treatments significantly (p < 0.001) decreased the average exposure to hyperglycaemia as measured by AUC for glucose values above 7.8 mmol/l and above 11 mmol/l, with a significant (p ≤ 0.01) treatment difference favouring exenatide (figure 2C and D, Table 2). Compared with baseline, both exenatide and sitagliptin significantly (p < 0.001) increased the time spent with glucose values between 3.9 and 7.8 mmol/l with a significant (p < 0.001) treatment difference favouring exenatide at endpoint (Table 2).

### Breakfast Test Meal

**Figure 3:**
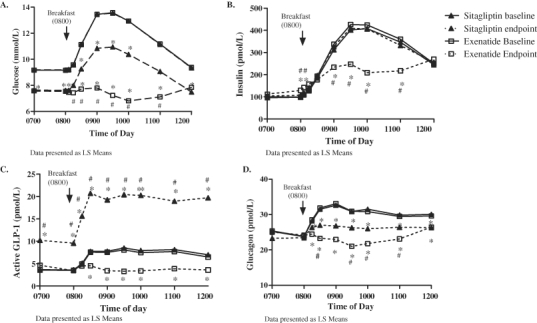
Glucose (A), insulin (B), intact GLP-1 (C) and glucagon (D) concentrations before and after individualized morning meal at baseline and endpoint. ^*^p < 0.005 change from baseline, ^#^p < 0.005 between-group comparison. GLP-1, glucagon-like peptide-1.

**Figure 4:**
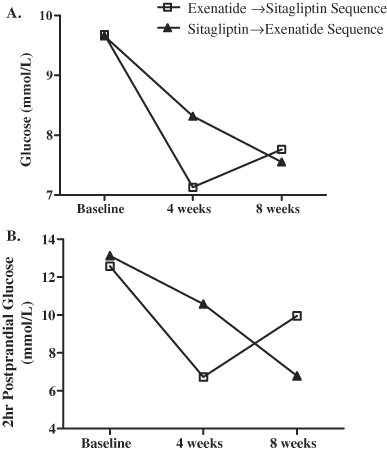
Metabolic parameters by sequence. (A) 24-h averaged glucose by treatment sequence at baseline, 4 weeks (end of treatment period 1) and 8 weeks (end of treatment period 2). (B) 2-h postprandial glucose by treatment sequence at baseline, 4 weeks (end of treatment period 1) and 8 weeks (end of treatment period 2).

At endpoint, the PPG concentrations were significantly lower (p < 0.005) with exenatide compared to sitagliptin at most time points following the test meal (figure 3A). Postprandial insulin concentrations with exenatide were lower at some points than with sitagliptin (figure 3B). At endpoint, postprandial concentrations of intact GLP-1 significantly increased over baseline with sitagliptin, but decreased with exenatide (figure 3C). While both exenatide and sitagliptin significantly (p < 0.005) decreased glucagon concentrations at most points after the breakfast meal, exenatide had a greater effect (p < 0.005; figure 3D). Figure 4A and B show the 24-h average glucose and the 2-h PPG at baseline, end of treatment period 1 and end of treatment period 2 by treatment sequence. The test for sequence (which can indicate a carryover effect) on the 24-h average glucose was not significant (p = 0.056).

### *β*-Cell Function

Both exenatide and sitagliptin significantly (p < 0.001) increased HOMA-B, with exenatide having a greater effect (p = 0.005, Table 2). At endpoint, fasting insulin levels were significantly higher in exenatide versus sitagliptin (135 vs. 105 pmol/l, respectively).

### Effect on Food Consumption

At baseline, the 24-h caloric intake (LS mean ± SE) was similar between treatment sequences (exenatide: 1968 ± 41 kcals; sitagliptin: 1952 ± 41 kcals). At endpoint, the caloric intake was significantly lower (p < 0.001) over 24 h in both groups, with exenatide having a greater effect (1745 ± 35 vs. 1853 ± 35 kcals; p < 0.001, respectively). At endpoint, the calorie intake was not significantly different between groups at the morning (exenatide 541 ± 15; sitagliptin 543 ± 15) or evening meal (exenatide 682 ± 17 kcals; sitagliptin 697 ± 17 kcals). Calories consumed were significantly less (p < 0.001) with exenatide compared to sitagliptin at the midday meal (exenatide 531 ± 18 kcals; sitagliptin 612 ± 18 kcals).

### Effect on Weight

Both treatments led to decreases in weight (−1.37 kg exenatide vs. −0.89 kg sitagliptin; treatment difference: −0.48, p < 0.05).

### Safety

The most common adverse events were nausea (exenatide 39%; sitagliptin 15%), vomiting (exenatide 19%; sitagliptin 5%), headache (exenatide 14%; sitagliptin 14%) and diarrhoea (exenatide 10%; sitagliptin 10%). Two subjects experienced serious adverse events (SAEs) while on exenatide (confusional state and anaphylactic reaction). Neither SAE was assessed to be related to the study drug. In the opinion of the investigator, neither event was related to exenatide treatment. No subject discontinued the study due to an adverse event. Three subjects on exenatide and one subject on sitagliptin experienced minor hypoglycaemia. There was no episode of major hypoglycaemia.

## Discussion

Several glucoregulatory mechanisms showed key differences between exenatide and sitagliptin in lowering blood glucose levels. Our study showed significant decreases in the average daily glucose, glucagon concentrations, caloric intake and improved *β*-cell function with exenatide treatment. The predominant glucodynamic difference between exenatide and sitagliptin in the current study was the observation that exenatide substantially decreased PPG concentration, with no significant difference on fasting glucose between the two drugs. This observation can be explained at least in part by multiple mechanisms, the most important of which may be glucagon suppression [15]. The current study showed that exenatide had a more pronounced effect on glucagon suppression than sitagliptin, which had a modest effect on glucagon. Greater glucagon suppression with exenatide compared to sitagliptin is consistent with a previous study [10]. Shah et al. demonstrated that lack of glucagon suppression can cause substantial hyperglycaemia when insulin availability is limited [15]. Cervera et al. showed that suppression of glucagon secretion and stimulation of insulin secretion by exenatide each accounted for approximately one-third of the decline in glucose concentrations following a meal, while the remainder of glucose suppression was because of delayed gastric emptying and increased splanchnic glucose uptake [16]. Thus, decreased PPG concentrations can be mediated, at least in part, by exenatide's greater effect on gastric emptying compared with sitagliptin [10]. Using acetaminophen absorption, DeFronzo et al. demonstrated significantly slower gastric emptying with exenatide than sitagliptin [10]. In the current study, subjects receiving exenatide consumed significantly fewer calories over a 24-h period compared to sitagliptin. This finding is consistent with that of DeFronzo et al. in which decreased caloric intake was observed with exenatide compared to sitagliptin during a test meal in a subset of patients [10]. The difference in caloric intake occurred at the midday meal, with the exenatide group consuming significantly fewer calories than the sitagliptin group. One might have expected this difference to occur at the standardized breakfast meal or at the dinner meal because the injections were given then. However, the difference in calories between groups at the midday meal could be attributed to delayed gastric emptying and satiety occurring post breakfast when patients received exenatide before the breakfast test meal. Although we measured weight in the current study and a statistical difference was observed between groups, the study design and short duration preclude the ability to make clinical inferences.

Exenatide has been reported to improve measures of *β*-cell function in type 2 diabetes [17,18]. In the current study, exenatide had a greater effect on *β*-cell function than sitagliptin, as measured by HOMA-B (consistent with Defronzo et al. [10]). Decreased postprandial insulin concentrations in the current study during exenatide treatment compared to sitagliptin are consistent with other studies [18] and reflect improved *β*-cell function relative to the improved PPG concentrations.

A recent open-label study by Arnolds et al. showed no difference in PPG excursions over 6 h between exenatide and sitagliptin when either drug was added to treatment for 48 subjects with type 2 diabetes receiving insulin glargine [19]. However, several study differences exist between our study and that of Arnolds et al. We examined glucose over an entire 24-h period in the absence of insulin glargine. In Arnolds et al., the power to detect differences was much lower compared to our study, given only 15 and 16 completers in the exenatide and sitagliptin arms, respectively, and the parallel design of that study. Furthermore, Arnolds et al. reported fasting glucose concentrations well below those typically reported in clinical trials in which glargine was uptitrated [20–22]. The current study may be more typical of patients with type 2 diabetes in clinical practice.

The current study confirms previous observations that sitagliptin increases endogenous intact GLP-1 in subjects with type 2 diabetes [23]. In contrast, exenatide decreased postprandial intact GLP-1 concentrations. To our knowledge, this study is the first to report an apparent suppression of postprandial GLP-1 concentrations during treatment with exenatide. Such a reduction could be explained by a negative feedback mechanism whereby exenatide inhibited further GLP-1 release from L-cells. Flint et al. demonstrated that an intravenous infusion of GLP-1 suppressed GLP-2, which is also secreted by the L-cells [24]. Defronzo et al. showed that the molar concentrations of exenatide exceed those of GLP-1 during treatment with sitagliptin [10], which may account for the greater effect of exenatide on postprandial and 24-h glucose concentrations.

A limitation of this study is the lack of a washout period between the two treatments. However, the short half-lives of sitagliptin and exenatide (12 and 2.4 h, respectively) make a carryover effect unlikely [2,3]. Furthermore, the treatment difference in period 1 was greater than in period 2, so in the presence of a carryover effect, the treatment difference estimate would be biased towards showing no difference. Residual biologic activity of either drug cannot be ruled out. Strengths of this study include its double-dummy, double-blinded, crossover design in which all completing subjects received both drugs, with the subjects each receiving two injections and one capsule each day. This design limited potential study bias.

In conclusion, the 24-h glucose profiles obtained during treatment with either exenatide or sitagliptin revealed that the predominant difference between the drugs lies in the ability of exenatide to substantially decrease PPG concentration. The effect of exenatide to improve 24-h glucose profile relative to sitagliptin may be explained, at least in part, by a greater effect of exenatide on suppressing PPG concentration, decreased appetite, decreased caloric intake, and improved insulin response to the ambient glucose. The overall effects of exenatide in the current study may be because of a greater molar concentration of exenatide compared to the molar concentration of intact GLP-1 during sitagliptin therapy [10].
